# Vitamin D role in hepatitis B: focus on immune system and genetics mechanism

**DOI:** 10.1016/j.heliyon.2022.e11569

**Published:** 2022-11-15

**Authors:** Arghavan Asghari, Fatemeh Jafari, Maryam Jameshorani, Hossein Chiti, Mohsen Naseri, Anahita Ghafourirankouhi, Omid Kooshkaki, Alireza Abdshah, Negin Parsamanesh

**Affiliations:** a Infectious Diseases Research Center, Birjand University of Medical Sciences, Birjand, Iran; b Birjand University of Medical Sciences, Birjand, Iran; c Radiation Oncology Research Center, Iran Cancer Institute, Tehran University of Medical Sciences, Tehran, Iran; d Department of Radiation Oncology, Cancer Institute, Imam Khomeini Hospital Complex, Tehran University of Medical Sciences, Tehran, Iran; e Zanjan Metabolic Diseases Research Center, Zanjan University of Medical Science, Zanjan, Iran; f Department of Internal Medicine, School of Medicine, Zanjan University of Medical Science, Zanjan, Iran; g Department of Immunology, Faculty of Medicine, Birjand University of Medical Sciences, Birjand, Iran; h Cellular and Molecular Research Center, Birjand University of Medical Sciences, Birjand, Iran; i Zanjan University of Medical Science, Zanjan, Iran; j School of Medicine, Tehran University of Medical Science, Tehran, Iran

**Keywords:** Vitamin D, Hepatitis B, Immune system, Genetics

## Abstract

According to the World Health Organization (WHO) report, viral hepatitis has been a problem in human society. Vitamins play a significant role in preventing the hepatocarcinoma and liver cirrhosis. In this report, we will first focus on the vitamin D function in the immune system reactions, and then investigate its role in the viral infections and the signaling pathway of hepatitis B virus.

The existence of the cytochrome P450 (*CYP) 27B1* enzyme, which is involved in vitamin D synthesis in immune system cells, has drawn researchers ' attention to the field of immune system. Toll like receptor (TLR) play a significant role in the immune system, and are one of the primary receptors of the innate immune system. In addition, the synthesis of inflammatory cytokines, such as Interferon γ (IFNγ) and Interleukin-2 (IL-2) is one of the key roles of T helper type 1 (Th1) cells; these cells can suppress two cited cytokines via vitamin D. In the chronic phase of hepatitis B, Cytotoxic T lymphocytes (CTLs) cells have weaker performance than the acute phase of the disease. The association between vitamin D physiologies with viral infections is also confirmed by genetic studies, carried out on genetic variations of vitamin D receptor *(VDR)* R-encoding disease susceptibility gene. Vitamin D affects different phases of the disease. Therefore, further experiments in this area are proposed.

## 1 Introduction

Viral hepatitis has become a problem in human society. According to the World Health Organization (WHO) report, one third of people suffer from hepatitis B (HBV) and hepatitis C viruses (HCV) [1, 2]. Hepatitis B has infected one-third of the world's population, 5% of infected individuals are known as carriers; among the infected subjects, 25% have chronic liver inflammation, which progresses to liver cirrhosis and finally hepatocarcinoma [3]. Diet plays an important role in counteracting the effects of hepatocarcinoma and liver cirrhosis; one of the reasons could be the presence of vitamins in the diet [4, 5]. Vitamins affect the development of hepatocarcinoma and liver cirrhosis [6].

1, 25-Dihydroxvitamin D3 (1, 25 (OH) D3) or vitamin D is one of the fat-soluble vitamins. It is mainly produced through UV radiation and conversion of 7-dehydrocholesterol to 1,25(OH)D3; although this vitamin is also obtained through diet such as: Oily fish, meat, milk products and fortified food products [7, 8]. Generally has been agreed, that the least serum/plasma concentration of 25-hydroxyvitamin D (25(OH)D) is approximately 30 nmol/L to prevent vitamin D deficiency-related bone disease; hence this threshold is appropriate for defining vitamin D deficiency in population - based studies. Vitamin D deficiency has become a global issue in the 21st century. According to the existing studies, vitamin D deficiency has been seen in one billion people worldwide [9]. Vitamin D status differs in various populations. Multiple studies have reported the rate of vitamin D deficiency with an evaluated prevalence of 30–93% in population with strong sun exposure, such as China, Turkey, India, Iran and Saudi Arabia over the past twenty years [10]. Recently, vitamin D have been introduced as an effective factor in immune system regulation, autoimmune diseases, cardiovascular and respiratory health, pregnancy, obesity, erythropoiesis, diabetes, muscle function, and aging [11].

Vitamin D can show its effect on the immune system directly and indirectly. It directly modulates and regulated the immune system [12, 13]; but indirectly has special effects on the progression and treatment of infectious disease. For example, it affects the absorption of minerals, such as calcium. It also has synergic effect with other vitamins and mineral, like zinc and vitamin C; each specifically affects the immune system [14, 15]. They makes it difficult to understand the main function of vitamin D in antiviral drugs [16, 17]. Studies have shown that vitamin D deficiency can reduce the effectiveness of antiviral drugs [17, 18].

In this article, we will first focus on the vitamin D biosynthesis and signaling in the immune system responses (innate and adaptive), and then discuss its role and effects on HBV immune-pathogenicity, due to viral infections and involved polymorphisms.

### 1.1 Vitamin D biosynthesis and signaling

A large body of evidence has suggested, that vitamin D could be obtained either from the diet of individuals or through synthesis in skin following exposure to sunlight. Because of cutaneous production of vitamin D due to Ultraviolet B (UVB) exposure, its synthesis might be related to different individualized factors, such as: latitude, season, use of sunblock, and skin pigmentation. Melanin has a key role in the synthesis of vitamin D; it absorbs the UVB radiation and inhibits Vitamin D synthesis from 7-dihydrocholestrol. As this form of vitamin D is inactive, in the next step it is hydroxylated in the liver to form 25(OH)D3 [19, 20].

It should be noted that this form of vitamin D (25 D) is still inactive, and mostly is used as a reliable form to measure and evaluate Vitamin D levels in individuals. This form 25(OH)D3 could be activated in kidneys with the effect of 1-alpha-hydroxylase, an enzyme which could be simulated by parathyroid hormone (PTH); it converts to active compound of vitamin D, named 1, 25 dihydroxy vitamin D (1, 25 D) or calcitriol [21, 22].

This activated form (1, 25 D) could be further metabolized in the liver to produce the inactive form of 1, 24, 25 vitamin D, by mediating 24-hydroxylase enzyme (*CYP24A1*). In the physiological condition, a negative feedback loop regulates the levels of 1, 25 D in blood circulation. In this regard, the circulating level of the active form of vitamin D is maintained within limited boundaries, through negative feedback loop; 1, 25 D could inhibit renal 1-alpha-hydroxylase and stimulate the 24-hydroxylase enzyme. In addition, 1, 25 D has a couple of roles; it induces calcium absorption in the intestine, and osteoblast differentiation and matrix calcification in the bones. The active form implicates in the normal function of these organs by binding to *VDR* [7].

Vitamin D insufficiency in chronic liver disease is thought to be multiple [23]. A decreased liver function might be one explanation for the low vitamin D levels seen in chronic liver diseases. Hepatic damages result in lower synthesis of vitamin D carrier proteins such vitamin D binding protein and albumin, as well as reduced vit D hepatic hydroxylation to 25(OH)D3 or calcidiol [24]. In a review published in 2012, Stokes et al. defined the role of vit D in liver disease extremely effectively [25]. Decreased exogenous exposure of patients to vit D sources, dietary vitD3 intestinal malabsorption, poor hepatic hydroxylation of 1, 25(OH)D3 to 25(OH)D3, and enhanced catabolic elimination of 25(OH)D3 are reported as key factors for vit D insufficiency in liver abnormalities [25]. Trepo et al [26], Wong GL et al [23], and Finkelmeier et al [27] have all demonstrated a link between liver-related complications and low Vit-D levels [28].

Mousa et al demonstrated that despite prescription vitamin D supplementation in high-dose and comprehensive endpoint assessments, vitamin D supplementation could not increase insulin secretion or sensitivity in vitamin D–insufficient, obese, or overweight patients. As a result, even in vitamin D-deficient communities, vitamin D supplementation is unlikely to be an effective approach for lowering diabetes risk (clinicaltrials.gov: NCT02112721) [29]. Vitamin D supplementation in childhood decreases the incidence of T1DM later in life, according to observation data obtained from case-control and some few cohort research [30, 31]. Clinicaltrials.gov recognized two RCTs (not yet published) on the prevention of asthma exacerbations in children (identifiers: NCT02687815 and NCT03365687) that may bring extra data on the effect of vitamin D in asthma cases [32].

**Figure 1:**
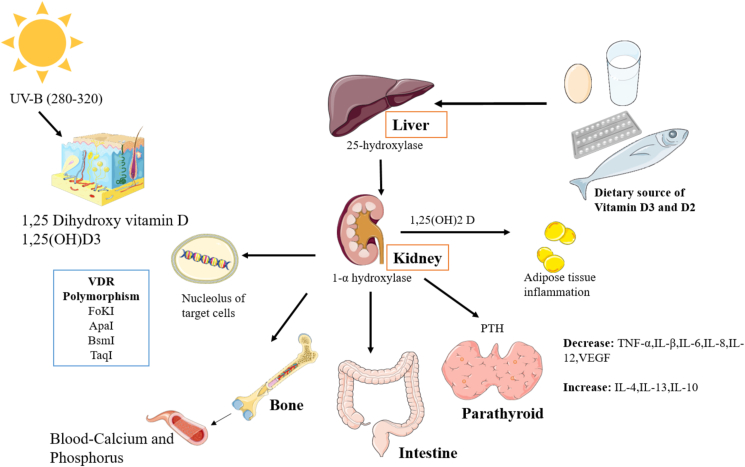
Vitamin D activation and metabolism.

*VDR* complex with the retinoid X receptor (*RXR*). The 1,25D-*VDR*-*RXR* heterodimer translocate to the nucleus, where it binds to the vitamin D responsive elements in the promoter regions of vitamin D responsive genes; it induces expression of these vitamin D-responsive genes. In addition, it has been shown that *VDR* could present on other tissues, including breast, bone marrow, brain, colon malignant cells and immune cells. For this reason, Vitamin D may have other important functions except its role in calcium and bone homeostasis. Furthermore, there are tissues other than kidney, which could express 1-alpha-hydroxylase; 25 D could be converted to 1, 25 D in these organs [33, 34]. Recently, Vitamin D is considered to have endocrine functions, as it could act in paracrine or autocrine manner. In this regard, Vitamin D has other different actions, including stimulation of cell proliferation and promotion of cell differentiation. It may have a substantial role in promoting the protective immunity by its immunologic effects (Figure 1).

### 1.2 Vitamin D supplementation in different conditions

Various factors such as age, weight, concomitant diseases, latitude, breed and nutritional culture can affect the daily requirement of vitamin D. Vitamin D supplementation should have no side effects, in addition to providing adequate serum levels of 25 (OH) D [35]. Most early studies examining the relationship between serum vitamin D level and individuals' health, considered 20 ng/ml as the minimal level of serum 25 (OH) D for human well-being; but it has been contested by recent researchers. Most recent studies have found that maintaining the serum level of 25 (OH) D in the range of 30–50 ng/ml or 40–60 ng/ml is more appropriate [35, 36, 37].

According to the Australian research, the minimum serum level of this vitamin to reduce the risk of skeletal diseases, such as rickets (10 ng/m) and osteoporotic fractures (20 ng/ml) appears lower, in comparison to the prevention of premature death (30 ng/ml) and non-skeletal diseases, such as depression (30 ng/ml), diabetes and cardiovascular disease (32 ng/ml), falls and respiratory tract infections (38 ng/ml) and cancer (40 ng/ml) [38].

When there is vitamin D deficiency, the European guidelines have recommend vitamin D supplementation as follows: 1000 IU/Day for neonates under one month, 2000–3000 IU/Day for infants over one month and toddlers, 3000–5000 IU/Day for children and adolescents aged 1–18 years, 7000–10000 IU/Day or 50000 IU/Week for adults and the elderly [39, 40].

In patients with vitamin D deficiency, who suffer from a concomitant disease, the need for vitamin D supplements may vary [41]. For example, in patients with intestinal malabsorption, either intramuscular injection of vitamin D or larger oral doses up to 50,000 IUS should be administered every 2–3 days; even UVB light can be used as an alternative treatment [42]. In severe hepatic and renal insufficiency, vitamin D deficiency should be treated with the active form of vitamin D) calcitriol (. However, in patients with chronic renal failure, vitamin D usage is important to maintain serum 25 (OH) D levels above 30 ng/ml [43]. In granulomatous diseases such as sarcoidosis and lymphoma, excessive vitamin D replacement will lead to hypercalcemia and osteomalacia. Serum 25 (OH) D levels should be maintained 20–30 ng/ml, and should not exceed 30 ng/ml [44]. Vitamin D deficiency should be treated in patients with hypercalcemic disorders, such as primary or tertiary hyperparathyroidism; in these patients vitamin D replacement to achieve at least 30 ng/ml of concentration, will not exacerbate hypercalcemia [44].

The maximum tolerable amounts of vitamin D intake, which do not lead to side effects are as follows: 1000 IU/Day for neonates under one month, 2000 IU/Day for infants and children between one month and 10 years, 4000 IU/Day for children and adolescents aged 11–18 years, 10000 IU/Day for adults and the elderly [45, 46]. The serum 25 (OH) D concentration up to 100 ng/ml is usually safe; the concentrations more than 150 ng/ml are considered toxic [47].

Glutathione (GSH), a tripeptide found mainly in the liver, is the most essential thiol decreasing agent essential in redox process regulation [48, 49]. Glutathione therapy has been shown quickly increase the anti-oxidative stress and improve the liver membrane structure in the treatment of chronic hepatitis B [50]. Moreover, GSH inhibits cytokine production, reduces active effector cell aggregation, prevents effector cell activation, and reduces cytokine induced damage, all of which contribute to cytokines' pro-inflammatory action on liver cells [51]. Glutathione is essential for the biosynthesis and hydroxylation of 1, 25-dihydroxy-vitamin D and 25-hydroxy-vitamin D from diary vitamin D in the body [52]. Also, in the livers of obese mice given a high-fat diet, glutathione deficiency causes epigenetic changes in vitamin D metabolism genes. Several reports have demonstrated that combining vitamin D with L-cysteine (a glutathione precursor) results in a more effective rise in circulating 25-hydroxyvitamin D and a reduction in inflammatory biomarkers [53].

In human renal proximal tubule epithelial cells, the increase in GSH level mediated by L-cysteine supplementation was associated with upregulation of VD-regulatory genes including *VDR, RXR* and *CYP27B1* and also downregulation of *CYP24A1*. Other studies confirmed that, supplementing with a combination of L-cysteine and VD or glutathione precursor, rather than just VD, is helpful and aids in the effective implementation of VD administration [54]. Moreover, combining vitamin D and LC supplementation may be more beneficial in reducing the risk of oxidative stress and adverse effect related with type 2 diabetes or COVID-19 infection [55, 56, 57]. This attitude be able to reduce cellular damage in systemic inflammatory disease, such as hepatitis, obesity, diabetes, and hypertension caused by cytokine storm. It appears that people with hepatitis B who have low glutathione levels need a different way to increase their vitamin D levels, immune system, and function in hepatitis B [58].

## 2 Role of vitamin D in immune system

### 2.1 Innate immunity and vitamin D

A complex network compromising many cells, tissues, organs, and the substances, which helps the body fight infections and diseases, while maintaining tolerance to self is named immune system. The immune system includes white blood cells and organs and tissues of the lymph system, such as the thymus, spleen, tonsils, lymph nodes, lymph vessels, and bone marrow [59]. The importance of vitamin D in the immune system was discovered, when the researchers found the role of vitamin D receptor and *CYP27B1*enzyme, which is involved in the vitamin D metabolism in the immune cells [60].

There are a couple of immune cells presenting *VDR* on the surface, including: antigen-presenting cells (macrophages and dendritic cells), T cells and B cells; on the other hand, the mentioned cells could synthase vitamin D. Additionally, it has been demonstrated that local levels of 1, 25 D may differ with circulating levels, as the mediating enzymes in local synthesis of vitamin D are different from the controls originating from kidney [61, 62].

Its regulatory role in the immune system was first considered by researchers, which is done by activating macrophages and monocytes [63]. The presence of *CYP27B1* enzyme, has attracted the attention of researchers to the field of immune system [64]. It should be noted that the extra renal 1-alpha-hydroxylase enzyme in macrophages is not the same as enzyme renal-hydroxylase, as it not regulated by PTH [65]. Extra renal hydroxylase function depends on circulating levels of vitamin D, rather it could be stimulated by cytokines, such as IFNγ, IL-1, or tumor necrosis factor (TNF-α) [66]. Moreover, the macrophage 24- hydroxylase enzyme is a nonfunctional splice variant, so there is no negative feedback to regulate the local levels of 1, 25 D [67, 68].

Researches have shown, that vitamin D can play a significant role in both innate and acquired immune systems [69, 70]. TLRs are stimulated by PAMPs (pathogen associated molecular patterns) and DAMPs (damage associated molecular patterns) detection; they induce immune responses against infectious agents including fungal, bacteria and virus [71]. This receptor initiates anti - viral reactions by detecting the nucleic acids and envelope glycoproteins; antiviral response involves the production of cytokines, interferon and chemokines [72].

Vitamin D can reduce TNF-α production by affecting TLR 2 and 4 expression [73, 74]. A study of vitamin D levels and TLR3 receptor gene expression found, that vitamin D reduces receptor expression in the epithelial cells [75]. In another study by Alvarez-Rodriguez et al., 25OHD form of vitamin D reduced the TLR7 receptor expression in acquired immune cells and monocytes [76]. Djukic et al. showed, that vitamin D deficiency reduced the levels of proinflammatory cytokines by TLR1/2, TLR3, TLR4 and TLR9 [77]. Although vitamin D appears to have a decreasing effect on the TLR genes expression, some studies have reported conflicting findings. For example in a study by Samar Ojaimi and colleagues in 2013, it was shown that after taking vitamin D and reache a sufficient level in the vitamin D deficient individuals, TLR 2 gene expression has greatly increased [78].

Stimulation of innate immune receptors by DAMP and PAMP causes antimicrobial peptides expression. Antimicrobial peptides with at least 100 amino acids in their structure are considered as antimicrobial agents in the innate immune system. In general, antimicrobial peptides are divided into two categories: cathelicidin and defensins [79]. Although we are more likely to recognize this infectious antimicrobial peptide as a defense barrier against bacteria, their role in the viral infections has also been observed, recently [80, 81].

LL-37 is the only member of the human cathelicidin family, overall LL-37 is effective in regulating immune system, and has spectrum antimicrobial activity role. Studies have shown that LL-37 binds to dsRNA and has antiviral activity [82, 83]. LL-37 binding to RNA, causes LL-37 detection by scavenger receptors (SRs), and enhances TLR3 signaling, then generates an inflammatory response [84, 85]. A study found that the presence of vitamin D induced LL-37, reduces respiratory system infection [86]. Studies have shown, that vitamin D induces cathelicidin production by affecting signaling TLR2/1 [87].

**Figure 2:**
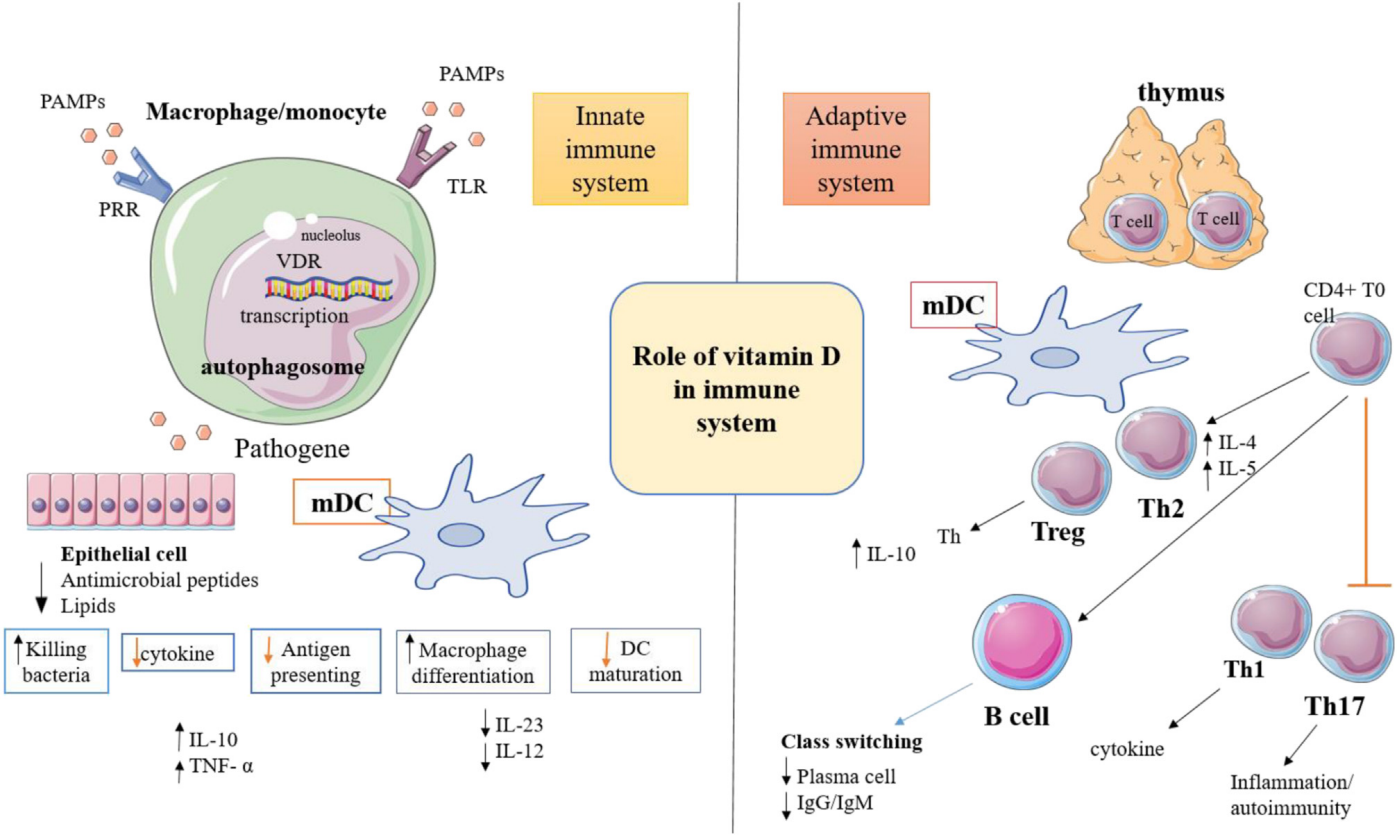
The role of vitamin D in the immune system.

Vitamin D also increases antiviral responses by inducing the expression of β-defensins [88]. Vitamin D levels can increase the expression of V β-defensin-2 and β-defensin-3 by stimulating the TLR2/6 receptors [89]. Some studies have shown that β-defensin-2 can cause antiviral responses by acting on virus receptors and replicating viruses [90] (Figure 2).

### 2.2 Adaptive immune system and vitamin D

Acquired immune system can be divided into two main parts: humoral immunity and cellular immunity. B lymphocytes are the main members of the humoral immune system. Cellular immune organs include T lymphocytes. In the following, we will examine the effect of vitamin D on these two systems. Despite the stimulant effect of vitamin D on innate immunity, it modulates the acquired immune system [91]. In the following, we will examine the effect of vitamin D on the humoral and cellular immunity.

Cellular immunity is a defensive immune mechanism involving the recruitment of phagocytes, antigen-sensitized cytotoxic T cells and the release of antigen-responding cytokines and chemokines. Cellular immunity is most powerful against the virus-infected cells, intracellular bacteria, fungi and tumor cells [92]. Important members of this immune system are T-cells, which themselves are generally divided into four basic sections, including T-helper 1, T-helper 2, T regulator, and cytotoxic T-cells; although these divisions are currently undergoing wide variations. One of the main functions of T helper (Th)1 cells is to produce inflammatory cytokines, such as IFNγ and IL-2, which can be reduced by vitamin D [92]. This property can be exerted by affecting the NFAT/AP-1 and Runx1 transcription factors [93]. But VitD3's direct impacts on Th2 cells are still unknown [94]. However, its effect on T-helper 2 cell is different from T-helper 1. It activates the cited cells and increases IL-4 production [91]. Vitamin D increases the function of regulatory T cells. This action is performed by increasing the transcription factor Foxp3, which has a special role in differentiating this cell from regulatory T cell [95]. Vitamin D also acts as a subset of other T-cells, T-helper 17 inhibits these cells. Inhibition of T-helper 17 function occurs in two ways: inhibition of IL-17 expression and IL-17 function suppression to differentiate T-helper 17 [93]. There are some studies, showed mDC treatment with vitamin D and taking it to a culture medium containing T cells, will increase the population of regulatory T cells [96]. In another study, it was found that vitamin D could directly affect regulatory T cells. A seasonal study of lymphocyte counts showed that the number of CD8 + and CD 4 + Tcells increases in summer [97]. Based on this, they hypothesized that vitamin D may affect T cell proliferation. One study showed that CTL activation affects the vitamin D receptors expression [98]. Some studies have shown, that vitamin D induces an inhibitory response to CTL and modulates the immune reactions in some infectious diseases [99, 100]. This is the case if it has a different effect on tuberculosis disease, and the presence of vitamin D causes activation of CTL and production of cytokines such as IFNγ and TNF-α [101]. Many studies have not been done on the effect of vitamin D on B cells. A study by Chen et al. showed, that B cells rarely act in the inactive state of *VDR*; activation of these cells are associated with this receptor expression increment [102]. Vitamin D appears to have an effect on antibody production and antibody class switching in the in vitro studies [102, 103]. But it does not show these effects in the in vivo studies [104]. On the other hand, vitamin D can indirectly inactivate B cells by affecting Tcells activation (Figure 2).

### 2.3 The role of vitamin D on HBV

According to existing studies, there are conflicting data on the effect of vitamin D on hepatitis B disease. Some studies have declared vitamin D deficiency reduces the therapeutic effects in infectious patients. Vitamin D inhibits virus replication [105, 106, 107, 108]; it has an effective role in platelet and albumin levels and reducing ALT enzyme levels in patients with acute hepatitis [109, 110, 111, 112]. In a research study, severe vitamin D deficiency in patients with autoimmune hepatitis has led to disease progression and lack of response to treatment. Severe vitamin D deficiency has been identified as a prognostic marker [113]. Another study of people with hepatitis B, which included patients with hepatocarcinoma and liver cirrhosis and hepatitis B infected individuals, evaluating the effect of vitamin D on disease progression, showed that the vitamin inhibits the adverse effects of disease and can prevent disease progression [105]. A prospective cohort study found that vitamin D deficiency was common in individuals with chronic HBV infection, and it was associated with adverse effects in the patients [23]. A study of HBV-transfected cells showed lowering the binding level of vitamin D increases the expression of viral genes and virus replication [114].

Other available studies point to lack of link between vitamin D levels and prevention of disease progression. Some studies found lack of association between vitamin D levels with HBV viral infection [115, 116]. In another study, it was shown that, vitamin D levels has no effect on hepatic fibrosis and viral loaded virus in antigen e positive cases [117]. A study also showed that hepatitis patients, who received tenofovir disoproxil fumarate (TDF) plus peginterferon alfa-2a (PegIFN) treatment complained about no effect of vitamin D normal level on the treatment effectiveness [109].

The discrepancies between studies on vitamin D deficiency and its effects on the hepatic infection of patients can be due to differences in the viral genotypes. For example, a study by Huijuan Zhu et al. showed that genotype B virus is more affected by vitamin D [118]. This statement has been confirmed by other studies [119].

The HBV virus is a noncytopathic virus; liver damages are due to the host immune reaction against its own infected cells [120, 121]. Although it has been a long time since the discovery of HBV, the immune-pathogenicity is still unknown. The main cause of liver damage is due to immune system responses to virus replication [122, 123]. CTLs play a major role in immune-pathogenicity. However, due to the inefficiency of these cells in completely eliminating viruses, other immune cells such as TH, Natural killer (NK) and Neu contribute to generate non-specific inflammatory responses [124].

HBV infection like other diseases, has two main phases, the acute and the chronic phases. Almost 95% of immune-competent persons with acute hepatitis successfully eradicate the HBV. But at this stage, we see disease complications, such as inflammation and necrosis of hepatocytes. The mortality rate is estimated to be 0.5–1% in the acute phase [125, 126]. During acute HBV infection, the serum level of ALT and T cells infiltration increase. It also reduces the level of HBV serum antigens and viral load.

The acute phase is associated with the onset of the innate immune response. Innate immunity plays an important role in disease pathogenesis. TLR receptors are important member of this system; they play a dual role, by producing inflammatory cytokines and preventing the proliferation of viruses and causing damage to the liver. The side effects are very mild and usually have no symptoms in the patient [127]. Acute inflammatory responses usually lead to virus clearance. Therefore, it is preferable to create strong immune responses at this stage, to prevent the disease from becoming chronic [127].

In a study shown on HBV transgenic mice, all receptors were TLR3, TLR4, TLR5, TLR7, and TLR9. They are involved in inhibiting HBV replication by producing antiviral cytokines, especially INF type I and TNF-α (Isogawa, Robek et al. 2005). Although it is not clear which pattern recognition receptors detect the HBV; studies have shown that the core protein of the virus is detected by TLR 2 on macrophages, due to the arginine-rich domain (Cooper, Tal et al. 2005). There is much evidence that TLR2 plays a special role in initiating an immune response against HBV. In a study by Cooper et al., the receptor was activated by the HBc antigen to activate macrophages and produce pro-inflammatory cytokines. As a result, the disease remains in the acute phase. Vitamin D reduces the pattern recognition receptors expression, and as a result it reduces the inflammatory cytokines production [128]. Some studies have shown that vitamin D by affecting TLR 2 expression can increase receptor expression, which in turn can inhibit the HBV virus in the early stages of the disease. It has been reported that HBV can interfere with TNF-α signaling and cathelicidin by reducing the expression of vitamin D receptor gene, thereby inhibiting the immune system response [129].

A study conducted by Monika Merkle et al. in 2015 showed, that IL37 secreted by the activation of innate immune receptors may have protective effect against HBV [130] According to other studies on the effect of vitamin D on IL37, it is expected that vitamin D inhibits the disease by increasing the expression of this factor, but a study showed that vitamin D in hepatitis patients has no effects on IL37 [131].

CD14 is a glycoprotein that binds to some ligands such as LPS; it activates TLR 4. HBsAg binding to the CD14 receptor causes the innate immune response. Although studies have shown that CD14 is a LPS receptor, and responds to TLR 4. Due to its similar lipid structure to HBsAg, it also binds to CD14 (Vanlandschoot, Van Houtte et al. 2002). Studies have shown, that vitamin D increases the expression of CD-14 in epidermal keratinocytes and monocytes (Oberg, Botling et al. 1993, Schauber, Dorschner et al. 2007).

Many studies have been done on the effect of vitamin D on NK and T cells. But in general, it can be said that vitamin D has a direct effect on the evolution and proliferation of NKT cells; in the situation of vitamin D deficiency, the number of NKT cells decreases [132, 133, 134]. Analyzes of Peripheral blood mononuclear cells (PBMCs) in individuals in the acute phase of hepatitis B disease showed an increase in NKT count, and its activity before HBV-specific T cells activation [135]. These cells play an important role in controlling the acute phase of HBV by producing cytokines, such as IFNγ and TNF-α. These cytokines cause cccDNA (covalently closed circular DNA) instability and decrease serum HBV DNA levels [136].

The role of specific immune systems in the acute phase of hepatitis B disease can be attributed to T cells, especially CTLs. In the acute phase of hepatitis B disease, these cells generate polyclonal T-cell response, while in the chronic phase of the disease the monoclonal or even an undetectable T-cell response will be generated [137, 138].

In the acute phase of the disease, CTLs contribute immunopathological effects of hepatitis B by producing cytokines, such as IFNγ and cytotoxic activity [139, 140]. In general, it can be said that in patients with AHB, there is a disruption in the production of cytokines, such as type1 IFN I, IL15, and IFNγ. On the other hand, anti-inflammatory cytokines production, such as IL10 has been reported, which ultimately inhibits the immune system in the acute phase [136]. Vitamin D seems to strengthen this pathway by creating its anti-inflammatory responses and immunomodulatory effect in this phase.

The chronic phase of hepatitis B disease can be divided into four categories, including: immune-tolerant, immune clearance, inactive HBs antigen carrier and reactivation phase. The specifications of each phase can be seen in Table 1 [141, 142].

**Table 1:** Chronic phase of hepatitis B disease categories.

Category	HBV DNA	HBe Ag	Anti-HBe	ALT	
Immune-tolerant	High	Positive	Negative	Normal/low	Replication of HBV
Immune clearance	High (Decrease/Low)	Positive	Negative	high	Liver disease/inflammation
Inactive HBs antigen carrier	Low/Undetectable	Negative	Positive	Normal	Remission of liver disease
Reactivation phase	High	Negative	Positive	Elevated	

In the chronic phase of hepatitis B, CTL cells have weaker performance than the acute phase of the disease [143]. One of the causes of decreased CTL function in the immune-tolerant phase of chronic hepatitis B CHB may be an increase in the expression of inhibitory receptors, such as Cytotoxic T-lymphocyte antigen 4 (CTLA-4) and programmed cell death protein 1 (PD-1); they eventually cause the appearance of exhausted phenotype in these cells [144, 145, 146]. Studies have shown that vitamin D increases the expression of these two inhibitory receptors [147, 148]. Other function of vitamin D against CTL cells, in the chronic phase of hepatitis B disease, studies have shown that CTL cell depletion occurs [149]. Vitamin D can also inhibit the proliferation of CD8 cells [150].

In the immune-tolerant phase, the number of CD4 + CD25 + FoxP3 + cells increases, which ultimately suppresses the antiviral responses. Many studies have shown the special effect of vitamin D on these cells. It increases the number and the immune-inhibitory function of these cells [151].

One of the characteristics of the immune clearance phase in patients with chronic hepatitis is the increase of Th17 in the patients’ liver and blood [152, 153]. In patients with CHB, Th17 cells may be related to immune activation and disease aggravation [154]. Vitamin D has extensive inhibitory effects on Th17, which can be summarized as an inhibitory role of vitamin D in the differentiation, maintenance, transcription, and bioactivity processes [155].

In patients with CHB, the ratio of CD4+/CD8+ decreases [156]. However, a study by Zhang et al. showed that vitamin D can affect this ratio. In the study of HBV infected mice, IFN and vitamin D treated mice had significantly lower levels of liver enzymes (ALT &AST) after treatment compared to mice, which did not receive vitamin D. The level of CD4 + and CD4 +/CD8 cells increases; it causes a balance between the two populations of CD + 4 and CD+ 8 cells. The levels of cytokines, such as IFN-γ, TNF-α, and IL-2 decrease, while IL- 4 did not show any differences between the two groups [157].

Innate immunity has a special role in the immune-pathogenesis of CHB; it is considered as a target for treatment [158]. Many studies have shown that increased C-reactive protein (CRP) can lead to complications such as liver cirrhosis and fibrosis in the chronic phase of hepatitis B [159].

if the increase of vitamin D has a decreasing effect on CRP expression [160]. In CHB patients, NK cells have inhibitory phenotypes [161], but vitamin D has a dual effect on them. It increases the expression of stimulant receptors, such as NKp44, NKp46, and NKp30, and also KLR (CD158) expression [162]. Table 2 showed clinical trial studies of vitamin D and hepatitis B infection.

**Table 2:** Clinical trial studies of vitamin D and hepatitis B infection.

Study Title	Interventions	Locations	Detailed Description	Status	Current Primary Outcome
The Relationship Between Vitamin D and Hepatitis B Virus Replication	Dietary Supplement: Vitamin D	Taipei Tzu Chi Hospital, Buddhist Tzu Chi Medical Foundation	-Randomized case-control trial	Completed	- Dynamic Change of HBV DNA
		New Taipei city, Taiwan	-Total of 149 HBV carriers with inadequate vitamin D (<30 ng/mL)		- Change of Serum qHBsAg (IU/mL) [Time Frame: baseline, after 2-month vitamin D supplement]
			-Randomly divided to two groups: one group receiving vitamin D supplement (1600 IU/day) for 2 months and another group as controls		The serum qHBsAg levels were measured before and after 2-month vitamin D supplement
Oral Vitamin D Treatment for the Prevention of HCC	Drug: Vitamin D3	Randomized	-Potential participants will be identified from the follow-up cohort of chronic hepatitis B in the third	Not yet recruiting	-Change in serum levels of 25-hydroxy vitamin D [Time Frame: at baseline, and at 6 and 12 months]
			-The participant instructed to begin taking 2 tablets per day (800 IU total) of vitamin D3 besides their regular anti-virus treatment		-Change in serum levels of 25-hydroxyvitamin D at 6 months and 12 months compared to baseline
			-The researcher will investigate general treatment benefits and the potential to reduce the development of Hepatocellular carcinoma (HCC), also known as liver cancer		
The Beneficial Effect of Vitamin D Supplement to Peginterferon Alpha 2a or to Telbivudine Monotherapy in Patients With Chronic HBV Infection	Drug: Peginterferon + Vitamin D	Ziv medical center liver unit	Not Provided	Unknown	-Treatment efficacy [ Time Frame: 120 weeks ]
	Drug: Peginterferon	Safed, Israel, Israel			- Primary end point will be sustained viral response which was defined as clearance of HBeAg from serum and HBV
	Drug: Sebivo	Liver clinic			-DNA less than 10,000 copies/mL (2000 IU/mL) at 6 months after treatment
	Drug: entecavir + vitamin D	Safed, Israel			-HBsAg titre during treatment and at 6 months follow up will be measured
					-Histologic response [Time Frame: 120 WEEKS]
To Study the Effect of Adding on PEG-INF Therapy for Patients Diagnosed With Chronic Hepatitis B	Drug: PEG-IFN & Nucleos(t)tide analogues	King Abdulaziz Medical City	-IFN-α with its dual immunomodulatory and antiviral effects was the first drug for Chronic HBV treatment followed by introduction of nucleos(t)ide analogues (NA)	Unknown	The loss of HbsAg between groups (NA) group and NA + Peg_INF group assessed by HbsAg test [Time Frame: 48 weeks]
		Jeddah, Saudi Arabia	- Directly inhibit HBV polymerase and provide an effective on treatment maintained viral suppression		The loss of HbsAg between groups (NA) group and NA + Peg_INF group assessed by HbsAg test
		King Abdulaziz Hospital	- PEG-IFN allows a convenient once a week dosing interval and of equal or superior treatment efficacy than conventional (IFN)		
	Drug: Nucleos(t)tide analogues	Jeddah, Saudi Arabia	Due to its predominant immunomodulatory effect PEG-IFN offers the advantage of higher sustained off treatment response rate compared to NA thus allowing a finite duration of treatment. The NA act by directly inhibiting HBV polymerase resulting in effective on treatment maintained viral suppression (HBV DNA PCR <200 for last 3–6 month)		
		King Abdulaziz Medical City			
		Riyadh, Saudi Arabia			

### 2.4 Genetic studies polymorphisms

#### 2.4.1 The association between vitamin D with viral hepatitis

##### 2.4.1.1 Genetic studies polymorphisms

It is true that vitamin D levels can be an effective factor in disease pathogenesis, but various genetic factors can affect this process; they include the polymorphism of the involved enzymes and receptors in the signaling pathway. The relationship between physiology of vitamin D and viral infections is also supported by genetic researches; they evaluated effective variants in the disease susceptibility, which encoding in the *VDR* gene.

Earlier studies revealed that the human *VDR* is a 75 kb nuclear receptor gene, found in the long arm of chromosome 12; it is composed of 11 introns and 11 exons [163]. Vitamin D polymorphisms are different interindividual, and are dependent on the disease. Taiwan was the first country, which indicated the major effect of host genetic history on infection result [164]. Furthermore, genetic variations in TNF and IFN, vitamin D receptor, estrogen receptor-1, and multiple HLA loci have been related to CHB in the subsequent studies [165, 166]. Similarly, in various populations, HLA-DR 13 has been identified to have a protective role against chronic HBV infections. Recently, Kamatabi et al. showed that genetic variants of HLA-DP locus are substantially related to the risk of chronic HBV infection in Asian patients by genome-wide association strategy [167]. The HLA-DP complexes are implicated in the presentation of antigen, and notably have been identified as predictive factors after HBV vaccination for antibody development [167]. BsmI, TaqI, FokI and ApaI described by the endonuclease, are the most common studied SNP [168]. Studies have shown that *VDR* gene polymorphism in rs731236 (Taq-1) (tt genotype) is significantly lower in patients with hepatitis B (171). Other studies have shown, that polymorphism rs2228570 (FokI) genotype ff makes people more susceptible to hepatitis B and also hepatocarcinoma [169, 170]. Other studies have shown that rs7975232 (ApaI) polymorphism increases the liver damage risk caused by HBV [171]; this polymorphism is also a prognostic factor in the effectiveness of treatment of hepatitis patients with Peg-IFN monotherapy [172, 173]. In a study about the rs222020 variant, which is linked to the vitamin D-binding protein gene, Hbe-negative individuals who received Peg-IFN treatment were found to have normal levels of ALT liver enzymes, as well as lower Hbs antigen levels [174]. Gao and coworker demonstrated, that rs1540339, rs11168268, rs2239182, rs3819545, rs2239184, rs2239186 and rs7041 SNP sites were related to the susceptibility of healthy volunteers to the HBV infection [175]; also, rs1800871, rs11168268, rs1544410, rs1800872, rs731236, rs3733359, rs1800896, and rs7041 polymorphisms were associated with hepatocellular carcinoma. In addition, four SNPs (rs2239184, rs2239186, rs2239181 and rs11168268) were meaningfully related to decompensation in cirrhotic cases. Two SNPs of rs2239184 and rs2239186 were significantly observed in all phases, except HBV infection to hepatocellular carcinoma [175]. In chronic hepatitis B infection the frequency of polymorphism in TaqI, ApaI and BsmI alleles is related to HBeAg, directly [176]. The polymorphism involved in the CYP2R1 (rs12794714) gene can be involved in interferon-based therapies, known as PegIFN therapy [177]. Variation in ApaI, is often linked to increase HBV viral load and greater fibrosis and necroinflammation [178]. A study showed that *TaqI VDR* variation is often linked with both chronic HBV infection and occult HBV disease. In the cited study, negative HBsAg participants had lower probability of HBV load [179]. Variability in the BsmI SNPs allele frequency is significantly related to primary biliary cirrhosis, while variability in *FokI* variants is linked with autoimmune hepatitis [180]. TNF-α and TNF-β are formed by stimulated lymphocytes. They activate Nf-kB, and then induce hepatic fibrosis-related proinflammatory and hepatic inflammation genes [181, 182]. The A/A genotype of TNF-β (sited in intron 2 of the gene) is more frequent in patients with severe liver problem due to chronic hepatitis B, compared to moderate liver diseases. In patients with chronic HCV, TNF-β A/A mutations have also been known to be associated with more serious liver fibrosis and problems [183, 184]. According to the numerous reports, *TaqI, FokI, ApaI*, and *BsmI VDR* polymorphisms have association with HBV infection risk and HBV-related liver diseases progression. Several findings have focused on HBV pathogenesis and development of new therapies. The recent discovery of genetic variants in the IL-28B locus, may predict hepatitis C therapy for viral clearance [185, 186]. These current discoveries could translate into genotype-based therapy choices for HBV infected individual.

## 3 Conclusion

The relation between vitamin D physiologies with viral infections is also confirmed by genetic research, carried out on genetic variations of VDR R-encoding disease susceptibility gene. Vitamin D inhibits virus replication; it has an effective role in platelet and albumin levels and reducing ALT enzyme levels in patients with acute hepatitis. In most HBV patients, vitamin D deficiency has been identified especially in advanced liver diseases associated with adverse clinical outcomes. However, Vitamin D can play different roles in diverse phases of disease. Therefore, it is suggested to perform more researches in this field.

## Declarations

### Author contribution statement

All authors listed have significantly contributed to the development and the writing of this article.

### Funding statement

This research did not receive any specific grant from funding agencies in the public, commercial, or not-for-profit sectors.

### Data availability statement

No data was used for the research described in the article.

### Declaration of interest’s statement

The authors declare no conflict of interest.

### Additional information

No additional information is available for this paper.
